# Morbidity in children with major kidney anomalies: a European population-based study

**DOI:** 10.1007/s00431-025-06232-3

**Published:** 2025-06-10

**Authors:** Ester Garne, Mads Damkjær, Anke Rissmann, Clara Cavero-Carbonell, Mika Gissler, Amanda Neville, Michele Santoro, Joachim Tan, David Tucker, Maria Loane, Joan Morris

**Affiliations:** 1 Department of Paediatrics and Adolescent Medicine, Lillebaelt Hospital, University Hospital of Southern Denmark, Sygehusvej 24, 6000 Kolding, Denmark; 2 Medical Faculty, Malformation Monitoring Centre Saxony-Anhalt, Otto-Von-Guericke-University Magdeburg, Magdeburg, Germany; 3 Rare Diseases Research Unit, Foundation for the Promotion of Health and Biomedical Research in the Valencian Region, Valencia, Spain; 4 Department of Data and Analytics, THL Finnish Institute for Health and Welfare, Helsinki, Finland; 5 Centre for Clinical and Epidemiological Research, University of Ferrara and Azienda Ospedaliero Universitario Di Ferrara, Ferrara, Italy; 6 Unit of Epidemiology of Rare Diseases and Congenital Anomalies, Institute of Clinical Physiology, National Research Council, Pisa, Italy; 7 NIHR Great Ormond Street Hospital Biomedical Research Centre, UCL GOS Institute of Child Health, London, UK; 8 Data & Digital Directorate, Public Health Wales, Swansea, UK; 9 Institute of Nursing and Health Research, Ulster University, Belfast, UK; 10 School of Health and Medical Sciences, City St George’s, University of London, London, UK; 11 Department of Molecular Medicine and Surgery, Karolinska Institutet, Stockholm, Sweden; 12 Academic Primary Health Care Centre, Region Stockholm, Stockholm, Sweden

**Keywords:** Congenital hydronephrosis, Multicystic kidney dysplasia, Posterior urethral valves, Kidney failure, Kidney transplantation

## Abstract

Knowledge about the prognosis for children born with congenital anomalies is important for counselling parents after a prenatal diagnosis. Nine population-based European Congenital Anomaly registries provided data on all children born 1995–2014 diagnosed with congenital hydronephrosis, multicystic kidney disease (MCKD), or posterior urethral valves (PUV) and on reference children from the same populations. Data up to 2015 on prescriptions, hospital diagnosis, and surgical procedures up to the 10th birthday were obtained by linkage to prescription and hospital databases. The study included 5624 children diagnosed with congenital hydronephrosis, 1314 with MCKD, and 414 with PUV. Children with hydronephrosis or MCKD were 13 times more likely to have prescriptions for antihypertensives compared to reference children before 10 years of age. Around 3% of children with congenital hydronephrosis or MCKD had a diagnosis of kidney failure at the age of 5 years; however, only 1% had a kidney transplantation by the age of 5 years. For children with PUV, 18.3% had a diagnosis of kidney failure and 5% had a kidney transplantation by the age of 5 years. *Conclusion*: Children born with congenital hydronephrosis and MCKD generally have a low absolute risk of developing kidney failure. Children with PUV have much higher morbidity, with 1 in 5 (18.3%) being diagnosed with kidney failure before the age of 5 years. It is important to monitor these children closely in early childhood in order to prevent or delay kidney failure.

**Table:** 

**What is Known:** • *Congenital kidney anomalies are a leading cause of pediatric end-stage kidney disease. Children with hydronephrosis, MCKD, and PUV have increased morbidity, but long-term data on medication use and kidney outcomes are limited.*	
**What is New:** • *This population-based study shows a low absolute risk of kidney failure in children born with hydronephrosis or MCKD but a high relative risk. PUV has high morbidity, with 1 in 4 developing kidney failure by age 10. Antibiotic use is high in early childhood, and antihypertensive use increases with age.*

**What is Known:**

• *Congenital kidney anomalies are a leading cause of pediatric end-stage kidney disease. Children with hydronephrosis, MCKD, and PUV have increased morbidity, but long-term data on medication use and kidney outcomes are limited.*

**What is New:**

• *This population-based study shows a low absolute risk of kidney failure in children born with hydronephrosis or MCKD but a high relative risk. PUV has high morbidity, with 1 in 4 developing kidney failure by age 10. Antibiotic use is high in early childhood, and antihypertensive use increases with age.*

## Introduction

Congenital anomalies of the kidney and urinary tract have a wide range of severity from spontaneous resolving congenital hydronephrosis to lethal bilateral renal agenesis. Severe kidney and bladder anomalies may lead to kidney failure and end-stage kidney disease with a need for kidney transplantation, and these anomalies are the main reason for kidney transplantation in early childhood [1, 2].

Prenatal detection rates of kidney anomalies have increased in Europe over the recent decades, and now most of the anomalies are diagnosed prenatally [3, 4]. Despite earlier detection, the long-term prognosis for children with posterior urethral valves (PUV) seems to be unaffected by a prenatal diagnosis [4, 5]. Knowledge about the prognosis for children born with kidney anomalies and the risk of developing end-stage kidney disease is important for counselling parents after a prenatal diagnosis. The risk of developing end-stage kidney disease is reported to be from 0 to 5% for children born with congenital hydronephrosis [6), 2 to 3% for children with MCKD [7, 8], and is much higher for children with PUV (up to one third) [4, 5, 9]. Studies on children with end-stage kidney disease include children with different etiologies for their kidney failure [1, 2, 10, 11]. In addition, most studies report results based on children followed in tertiary centers, but results from population-based studies may give more valid data for counselling the parents as less severe cases may be included.

The EUROlinkCAT study analyzed morbidity for liveborn children with congenital anomalies by linking data from population-based EUROCAT congenital anomaly registries to hospital databases to obtain information on length of hospital stays and surgical procedures [12]. We have previously published that children with congenital hydronephrosis and MCKD had a median length of stay in hospital in the first year of life of 7.3 days and 4.6 days, respectively [13]. Less than half of these children had a surgical procedure performed before the age of 5 years [14]. For children born with PUV, morbidity was much higher, with a median length of stay in the first year at 20.3 days, and 86% had a surgical procedure performed before the age of 5 years [15].

The aim of this EUROlinkCAT study is to further describe morbidity for children born with congenital hydronephrosis, MCKD, and PUV based on the number of antibiotics and antihypertensives prescribed/dispensed and to describe the proportion of these children that had kidney surgery, kidney failure, and kidney transplantation.

## Material and methods

This study is a population-based data-linkage cohort study, including data from nine EUROCAT registries (national or regional) in six European countries (Table 1) PMID: 21384529, Paper 5: Surveillance of multiple congenital anomalies: implementation of a computer algorithm in European registers for classification of cases. Children with congenital hydronephrosis are included in EUROCAT if the pelvis diameter is 10 mm or more after the first days of life. Children with hydronephrosis secondary to vesico-ureteral reflux are not included in EUROCAT. All children born without a congenital anomaly from the same birth years and from the same geographical area covered by the registry were included as a reference population (reference children) [12, 13]. The registry in Tuscany used a 10% random sample of their population as reference children, and data on reference children were not available from the three English registries.

**Table 1:** Number of children with three kidney anomalies by registry

**EUROCAT registry**	**Birth years included**	**Reference children**	**Children with congenital hydronephrosis**	**Children with multicystic kidney dysplasia**	**Children with posterior urethral valves**
Denmark, Funen	1995–2014	100,748	125	41	9
Finland	1997–2014	911,679	1920	426	177
Italy, Emilia-Romagna	2008–2014	223,995	257	44	11
Italy, Tuscany	2005–2014	23,503	206	59	19
Spain, Valencian Region	2010–2014	168,563	440	36	19
UK, Wales	1998–2014	531,784	1500	299	111
UK, East Midlands and South Yorkshire	2003–2012	Data for reference children not available	801	234	24
UK, Thames Valley	2005–2013		208	82	22
UK, Wessex	2004–2014		167	93	22
Total number of children		1,960,272	5624	1314	414
Number of deaths in the first year (% of total)		3156 (0.2%)	104 (1.8%)	69 (5.3%)	34 (8.2%)
Children with isolated kidney anomaly (% of total)			4676 (83%)	1087 (83%)	NA

*NA* not available.

Data on prescribed/dispensed medications were obtained by linking only children born from 2000 to 2014 to local electronic prescription databases, which were only available for the years 2000 to 2015. The prescription databases included medications prescribed by a physician (Wales) or dispensed by a pharmacy (Denmark, Finland, Italy, and Spain). Medications given during hospital stays are not included. There was no medication data from the English registries. Data on selected medications (antibiotics with ATC code J01 and antihypertensives with ATC codes C03, C08, and C09) were analyzed.

Data on discharge diagnosis and surgical procedures during hospitalizations for all children up to the child’s 10 th birthday or end of 2015, whichever came earlier, were obtained by electronic linkage to hospital databases. This ensured that at least 1 year of follow-up after birth was available for each child. Data for the age group 5–10 years of age were available for six of the nine registries as only children born in 1995–2005 reached the age of 10 years before the end of 2015. Details of the methods used in the EUROlinkCAT study including the linkage methods, results, and missing data have been published elsewhere [12, 13]. Overall, the linkage success between databases was 97.5% for children with congenital anomalies and 95.2% for reference children. The children that were not linked were excluded from the analysis.

The diagnosis of kidney failure was based on hospital discharge diagnoses. The codes used to define kidney failure were 586, V45.1, V56, and 996.56 in ICD-9/ICD9-CM and N17-19, Z49, Z992, and P960 in ICD-10. These discharge codes include diagnoses of acute and chronic kidney failure including neonatal kidney failure and any hospital contacts due to dialysis. Surgeries were coded according to the coding systems used in the national health record systems. Italy and Spain used ICD-9-CM for the study period; Wales and England used OPCS-4, and Finland and Denmark used national adaptions of NCSP (NOMESCO Classification of Surgical Procedures). For this study, codes for kidney surgery and for kidney transplantation were defined. The definition of the codes for any surgery is described in more detail elsewhere [14].

The EUROlinkCAT study included all children born with one or more major congenital anomalies including those with a genetic diagnosis. The group of children in this study classified as having isolated kidney anomalies was based on the EUROCAT flowchart for the classification of multiple congenital anomaly cases which defines “isolated” as not having an additional anomaly from a different organ system nor having a genetic diagnosis [16]. As the study included aggregate data from each registry, there may be a few children with unilateral MCKD and unilateral hydronephrosis that are included twice. The results on kidney failure and prescribed/dispensed medications were available for children with isolated kidney anomalies only. The results on surgery, kidney transplantations, and mortality included all children with kidney anomalies and were not restricted to those with isolated anomalies. Due to a miscoding in the analysis of end-stage kidney disease, around 6% of children classified as having PUV actually had a diagnosis of Prune Belly without the diagnosis of PUV; the impact of this was examined in a sensitivity analysis as follows: It was assumed that 6% of children with Prune Belly had twice the kidney failure rate as those with PUV, enabling the failure rate for children with PUV to be estimated.

### Statistical methods

Individual case data were not available for analysis due to concerns about identifiability. Therefore, each registry provided summary results which were then combined using random-effects meta-analytic techniques. Firstly, within each registry, the proportion of children having kidney failure or surgery from birth to 1 year of age, from 1 year of age up to their 5 th birthday, and from birth up to their 5 th birthday were calculated using Kaplan–Meier survival curves to control for the varying lengths of follow-up of the children, mainly due to censoring the data at the end of the study. In addition, within each registry, the relative risk of kidney failure in children with kidney anomalies compared with reference children was calculated. Random-effects meta-analyses were used to provide overall estimates of these proportions and relative risks using the commands METAN and METAPROP in STATA v16. Random-effects models were used as the underlying hypothesis was that these anomalies increased the risk of mortality/kidney failure by the same amount in all children, but that there would be random fluctuations of this increased risk within each registry.

The overall proportion of children who had a prescription for an antibiotic or antihypertensive medication during each year of age was calculated within each registry, and then, a random-effects meta-analysis was performed to provide overall estimates of these proportions using METAPROP in STATA v16.

The median number of surgical procedures and the median age of surgery in the first 5 years reported by the registries were combined using the “metamedian” package in R, version 4.0.3. [17]. All other analyses were performed in STATA version 16.

## Results

### Demographics

The study included 5624 children diagnosed with congenital hydronephrosis, 1314 children with MCKD, and 414 children diagnosed with PUV. The distribution of cases per registry, number of reference children, and birth years included are presented in Table 1. For children with congenital hydronephrosis and MCKD, 83% were classified as isolated kidney anomalies.

### Prescription data

**Fig. 1:**
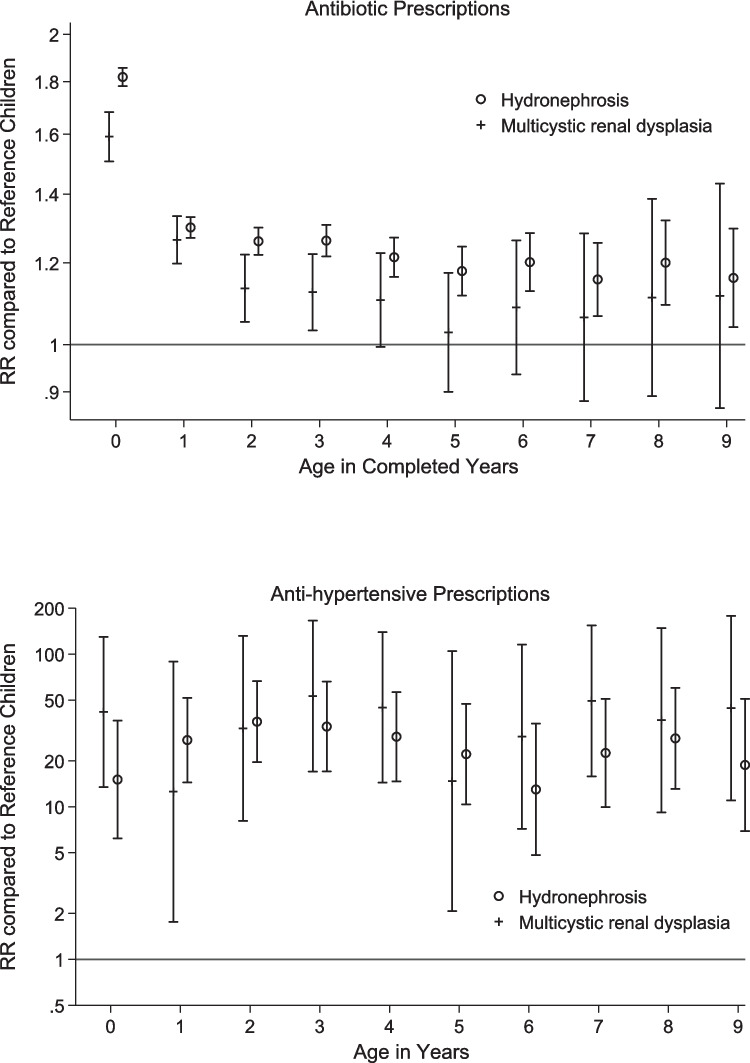
Relative risk of prescriptions of antibiotics and antihypertensives including diuretics for children born with hydronephrosis or multicystic kidney dysplasia

Among children with isolated congenital hydronephrosis and MCKD, 76% and 70%, respectively, received a prescription for antibiotics for outpatient use within the first year of life compared to 44% for the reference children (Table 2). The proportions were slightly higher in the second year for children with MCKD and for reference children. The proportion of children receiving a prescription for antibiotics per year decreased for the age groups 3–9 years. At age 9 years, 26% and 24% of the children with hydronephrosis and MCKD received a prescription for antibiotics, which was very similar to that for reference children (Fig. 1, relative risk).

**Table 2:** Percentage (95% CI) with prescriptions for children born with congenital hydronephrosis and multicystic kidney dysplasia by age groups, compared to children from the same populations without congenital anomalies (reference children)

	Age (completed years)	Congenital hydronephrosis		Multicystic kidney dysplasia		Reference children	
		Number	% with a prescription during the year (95% CI)	Number	% with a prescription during the year (95% CI)	Number	% with a prescription during the year (95% CI)
Antibiotics	< 1	3026	76 (73–79)	621	67 (63–71)	1,722,912	44 (40–48)
	1	2999	70 (68–72)	613	69 (62–76)	1,710,136	57 (52–62)
	2	2681	60 (59–62)	558	57 (48–65)	1,542,220	50 (46–55)
	3	2350	59 (55–63)	493	52 (44–59)	1,376,969	49 (44–53)
	4	2082	52 (45–58)	426	44 (35–53)	1,205,677	42 (39–46)
	5	1802	41 (36–46)	373	38 (31–46)	1,035,937	35 (31–39)
	6	1526	35 (29–41)	327	33 (25–42)	864,038	30 (26–34)
	7	1286	24 (17–32)	273	29 (23–34)	739,063	22 (18–27)
	8	1071	29 (26–32)	218	27 (21–33)	614,519	22 (19–25)
	9	916	26 (22–29)	187	24 (12–38)	532,411	20 (18–23)
Antihypertensive	< 1	3026	0.21 (0.07–0.41)	621	0.55 (0.09–1.32)	1,722,912	0.02 (0.01–0.03)
	1	2999	0.35 (0.17–0.60)	613	0.32 (0.00–0.97)	1,710,136	0.01 (0.01–0.02)
	2	2681	0.46 (0.23–0.75)	558	0.39 (0.01–1.13)	1,542,220	0.01 (0.01–0.02)
	3	2350	0.42 (0.19–0.72)	493	0.69 (0.11–1.66)	1,376,969	0.01 (0.00–0.02)
	4	2082	0.47 (0.22–0.82)	426	0.84 (0.15–1.98)	1,205,677	0.01 (0.01–0.02)
	5	1802	0.46 (0.19–0.83)	373	0.50 (0.00–1.54)	1,035,937	0.02 (0.01–0.02)
	6	1526	0.34 (0.10–0.71)	327	0.70 (0.03–1.98)	864,038	0.02 (0.01–0.03)
	7	1286	0.56 (0.21–1.05)	273	1.34 (0.25–3.12)	739,063	0.02 (0.01–0.03)
	8	1071	0.72 (0.28–1.32)	218	1.21 (0.10–3.20)	614,519	0.02 (0.01–0.04)
	9	916	0.51 (0.13–1.10)	187	1.41 (0.12–3.72)	532,411	0.02 (0.01–0.03)

The proportion of children receiving a prescription for antihypertensives including diuretics was low but increased with age. At age 9 years, 0.51% (95% CI 0.13–1.10) of the children with hydronephrosis and 1.41% (95% CI 0.12–3.72) with MCKD received a prescription for antihypertensives including diuretics compared to 0.02% (95% CI 0.01–0.03) for reference children. The risk of being prescribed/dispensed antihypertensives compared to reference children was over 13 times higher for all ages up to 10 years.

### Kidney failure and transplantation

Around 3% of children born with congenital hydronephrosis or MCKD had a diagnosis for kidney failure in the hospital databases, and the proportion was almost the same at ages 5 and 10 years (Table 3). Very few of these children had a record of kidney transplantation within the first 5 years of life (Table 4). For children with PUV, 18.3% have a record of kidney failure by 5 years of age, increasing to 26.1% by 10 years of age. Adjusting for the possible misclassification of 6% of children with Prune Belly syndrome having PUV resulted in an estimated 17% of children with PUV having kidney failure by 5 years of age. A code for kidney transplantation was present for 5% of these children by 5 years of age, with a median age at surgery of 136 weeks.

**Table 3:** Number and proportion of children with a hospital diagnosis of kidney failure according to congenital anomaly subgroup and age

Congenital anomaly subgroup	Kidney failure							
	**0–4 years**				**0–9 years**^**a**^			
	Total	*n*	Risk per 100 children^b^ (95% CI)	Relative risk compared to reference children	Total	*n*	Risk per 100 children^b^ (95% CI)	Relative risk compared to reference children
Reference children	1,960,272	366	0.01 (0.00–0.02)	1.0	1,544,211	432	0.03 (0.01–0.06)	1.0
Hydronephrosis^c^	4676	115	2.6 (1.6–3.9)	129 (107–155)	2940	77	2.9 (0.7–6.1)	90 (72–11)
Multicystic kidney dysplasia^c^	1087	31	3.0 (1.3–5.3)	153 (105–216)	619	14	3.3 (0.3–8.8)	82 (48–133)
Posterior urethral valves^c,d^	345	58	18.3 (10.3–28.0)	891 (667–1173)	246	46	26.1 (9.9–46.1)	645 (470–871)

^a^Only 3 registries included for 0–9 years

^b^1-Kaplan–Meier estimate of the measure of morbidity in age period from meta-analysis of all registries

^c^Children with isolated kidney anomaly

^d^Including < 5% with Prune Belly

**Table 4:** Kidney surgery and kidney transplantations for children born with congenital hydronephrosis, multicystic kidney dysplasia (MCKD), and posterior urethral valves

	Number of children born	< 1 year	1–4 years	< 5 years	**Median** number of surgical procedures in the first five years (**95% CI)**	**Median** age in weeks at first surgery in the first 5 years (**95% CI)**
**Percentage who had any surgery (95% CI)**^**a**^						
Hydronephrosis	5624	31% (25–36)	27% (23–31)	46% (41–51)	1.9 (1.6–2.1)	27.6 (19.6–35.6)
MCKD	1314	20% (15–25)	27% (18–37)	41% (30–52)	1.7 (1.3–2.1)	39.6 (25.7–53.5)
Posterior urethral valves	414	75% (67–82)	46% (36–55)	86% (81–89)	2.4 (1.9–2.8)	2.9 (1.7–4.1)
**Percentage who had kidney surgery (95% CI)**						
Hydronephrosis	5624	22% (16–27)	15% (13–17)	32% (27–38)	1.5 (1.2–1.9)	31.3 (23.1–39.4)
MCKD	1314	11% (7–16)	13% (7–22)	23% (16–27)	1.0 (1.0–1.0)	42.2 (26.0–58.4)
Posterior urethral valves	NA					
**Percentage who had kidney transplantation (95% CI)**						
Hydronephrosis	5624			0% (0–1)	1 (1.0–1.0)	168 (121–215)
MCKD	1314			1% (1–2)	1 (1.0–1.0)	148 (49–248)
Posterior urethral valves	414			5% (3–7)	1 (1.0–1.0)	136 (16–257)

^a^Previously published [8]

The results for kidney failure and kidney transplantation were similar across registries (not shown due to small numbers). Among the almost 2 million reference children followed up to 5 years of age, only 0.01% (95% CI 0.00–0.02) had a hospital diagnosis of kidney failure. Further, 1.5 million reference children were followed up to 10 years of age, of whom 0.03% (95% CI 0.01–0.06) had a diagnosis of kidney failure.

### Mortality

Mortality in the first year was 0.2% (95% CI 0.0–0.4) for children with isolated hydronephrosis and 2.3% (95% CI 1.2–3.4) for children with isolated MCKD compared to 0.2% for reference children. Mortality in the first year of life was higher for children who had other anomalies in addition to the kidney anomalies; for all children born with congenital hydronephrosis, mortality in the first year of life was 1.8% (95% CI 1.2–2.5%), 6.2% (95% CI 4.3–8.1) for all children born with MCKD, and 6.8% (95% CI 1.8–11.6) for all children born with PUV.

## Discussion

Our study showed that children with congenital hydronephrosis or MCKD received more prescriptions for antibiotics for outpatient use than reference children in early childhood. Urinary tract infection is a known risk for children born with hydronephrosis [6]. Also, children with unilateral MCKD and another anomaly on the other kidney are at increased risk for urinary tract infections with the potential impact on kidney function [18]. Febrile urinary tract infection in early childhood may result in kidney scarring [19]. Therefore, prophylactic antibiotics have been recommended for all children with congenital hydronephrosis, but more recent guidelines recommend antibiotics to be used for selected children during the first years of life only [6]. In our dataset, we do not know if the antibiotics were given for acute infections or as prophylactic treatment. We found a slightly lower rate of prescribed/dispensed antibiotics to children with MCKD compared to the children with congenital hydronephrosis.

Our study also showed a low but significantly increased proportion of children with MCKD compared to reference children having prescriptions for antihypertensives, which increased from the first year of life to age 9 years. A retrospective cohort study including children aged 0 to 18 (median 10) years of age with MCKD, single kidney, kidney hypoplasia, and PUV found that 19% were diagnosed with hypertension, and half of these children received antihypertensive medication [20]. The children with MCKD had the lowest risk of being diagnosed with hypertension (5%), and this seems to be comparable with the finding in our study of 1.41% being on medical treatment at age 10 years. As children with MCKD are at an increased risk of developing hypertension and end-stage kidney disease, they should be followed up during childhood according to the guidelines for children with a solitary functioning kidney [18, 21]. The risk of developing hypertension increases further as the children become teenagers and young adults.

We found a low absolute risk for having a hospital diagnosis of end-stage kidney disease for children born with congenital hydronephrosis and MCKD, although the relative risk was high compared to children without congenital anomalies. For children born with PUV, 1 in 5 had a diagnosis of kidney failure at 5 years, increasing to 1 in 4 at 10 years of age. The possible mechanism for developing end-stage kidney disease in children born with PUV is thought to be bladder dysfunction leading to obstructive uropathy and kidney dysplasia [4, 5]. Studies have shown that congenital kidney anomalies are the most common etiology for end-stage kidney disease and kidney transplantation in children [2, 10], but these studies do not report on the specific kidney anomalies. A Canadian study comparable to the methods in our study found that 31% of the children born with PUV developed chronic kidney disease within a median follow-up of 14.2 years [9]. This is in line with our results.

It is likely that the children in our study with congenital hydronephrosis and MCKD and a hospital diagnosis of end-stage kidney disease had bilateral kidney anomalies. A previous EUROCAT study for birth years 1997–2006 showed that among 421 liveborn children with MCKD, 361 (86%) had unilateral MCKD and 60 (14%) had bilateral MCKD [3]. There may also be children in our study with unilateral MCKD and a major anomaly of the contralateral kidney. As there is no clear definition of congenital hydronephrosis, it may be difficult to compare the proportion of children with end-stage kidney disease, as the prevalence in the populations studied may differ. EUROCAT has defined congenital hydronephrosis as a pelvis diameter of 10 mm or more after birth.

We do not know if the children with a diagnosis of kidney failure had end-stage kidney disease with the need for dialysis within the study period. However, it is likely that the diagnosis of kidney failure will progress to end-stage kidney disease later in childhood or early adulthood. Among children under 18 years of age undergoing kidney transplantation, only 20% were age 0–5 years, while 33% were age 6–12 years and 39% were age 13–17 years [11]. Children with end-stage kidney disease may stay on dialysis for longer periods before kidney transplantation takes place. However, long-term prognosis is best if the child undergoes transplantation just before the need for dialysis [11]. The period with dialysis is very challenging for the child and the family, with very frequent hospital contacts. It has also been shown that children and adolescents in rural areas with chronic kidney disease face unique challenges related to accessing pediatric nephrology care [22].

Prenatal detection rates for congenital hydronephrosis, MCKD, and PUV are high in Europe [3, 4]. Our results may be used in the counselling of parents after they receive the prenatal diagnosis. The prognosis for the majority of fetuses with congenital hydronephrosis is excellent with a low risk for developing kidney failure. The prognosis for fetuses with unilateral MCKD is also good, but parents should be informed about the follow-up program in childhood for children with solitary kidneys [18, 21]. The prognosis for fetuses diagnosed with PUV is less favorable with higher mortality, higher morbidity, and a high risk for developing end-stage kidney disease during childhood. These children should be followed in tertiary multidisciplinary teams.

Our study showed a very low risk of kidney failure among reference children with 1 per 10,000 children under the age of 5 years. These results are not directly comparable to other studies as other studies include children with kidney anomalies. For Europe, the prevalence of end-stage kidney disease for children under 5 years of age is reported to be around 20 per million in populations including children with kidney anomalies [2]. A population-based study from Canada comparing boys born with PUV with the general population of boys found a prevalence of chronic kidney failure of less than 0.1% in the general population, with a median follow-up of 16.6 years [9].

## Strengths and limitations

The main strength of our study is the population-based design including all children born in the registry areas with the specific three kidney anomalies studied. The study used the standardized case definitions from EUROCAT and further developed standardized linkage and analysis scripts for optimal data extraction from all linked databases in each region. The linkage success was very high. A limitation is that the study used aggregate data from each center due to confidentiality reasons. Therefore, a few children with congenital hydronephrosis in one kidney and MCKD in the other kidney may be included twice in this study and there may be a few children with PUV that also had a code for hydronephrosis. The diagnosis of kidney failure was based on discharge codes from the hospital databases. There may be differences across hospitals and doctors in how this is coded. It is also a limitation that information on medication prescribed/dispensed during hospital stays were not available for the study. However, we think that most children hospitalized with urinary tract infection were discharged before their treatment ended. Medical treatment for hypertension is chronic and will not be given only in hospitals. It is also a limitation that we do not know the indication for and type of antihypertensives prescribed/dispensed as ACE inhibitors may also be used for proteinuria. Data on prescriptions for children with PUV were not available as these data were only available for congenital anomalies with a livebirth prevalence of at least 1.75 per 10,000 births. The results on kidney failure/end-stage kidney disease were based on discharge diagnoses from the hospitals, and definitions may differ across countries and over time. We did not have access to the individual assessment of each child. However, it is reassuring that the proportion of children with a hospital discharge code for kidney failure was similar across registries. Unfortunately, we were not able to correct the error in the programming of end-stage kidney disease for children with PUV as we no longer had access to the linked hospitalization data when the error was found; hence, a small proportion of children with Prune Belly were included in this result. A sensitivity analysis to determine the possible bias of including around 6% of children with Prune Belly syndrome indicated that between 17 and 18.3% of cases with PUV would also have a code for kidney failure (assuming none of the children with Prune Belly had a code for kidney failure or that having a code for kidney failure was twice as likely for children with Prune Belly compared to those with PUV).

## Conclusions

Our study showed that children born with congenital hydronephrosis and MCKD generally have a low absolute risk of developing kidney failure, although the risk is higher than for reference children. Children with PUV have much higher morbidity with 1 in 4 being diagnosed with kidney failure before age 10 years. It is important to follow these children closely in early childhood in order to prevent or delay end-stage kidney disease.

## Data Availability

The data that support the findings of this study are available from the participating registries of congenital anomalies, but restrictions apply to the availability of these data, which were used under license for the current study, and so are not publicly available. Data are however availa-ble from the authors for scientifically valid requests and with permission of the participating registries of congenital anomalies.

## Abbreviations

ATC: Anatomical Therapeutic Chemical
EUROCAT: European Network for Surveillance of Congenital Anomalies
ICD: International Classification of Diseases
MCKD: Multicystic kidney dysplasia
PUV: Posterior urethral valves
